# Response to Letter to the Editor: “Comment on ‘Evaluating vaccination dosing strategies for SARS-CoV-2 in patients at high-risk for allergic reactions: Insights from vaccination campaign’”^☆^

**DOI:** 10.1016/j.waojou.2025.101118

**Published:** 2025-09-23

**Authors:** Stefania Nicola, Iuliana Badiu, Nicolo Rashidy, Elena Saracco, Erika Montabone, Luca Lo Sardo, Marzia Boem, Valentina Marmora, Federica Corradi, Andrea Ricotti, Richard Borrelli, Giovanni Rolla, Simone Negrini, Luisa Brussino

**Affiliations:** a Department of Medical Sciences, University of Turin, Immunology and Allergy Unit, Mauriziano Hospital, 10128, Turin, Italy; b Hospital Administration Unit, Mauriziano Hospital, 10128, Turin, Italy

Dear Editor,

We are grateful for the thoughtful comments on our article, “Evaluating vaccination dosing strategies for SARS-CoV-2 in patients at high-1 risk for allergic reactions: Insights from vaccination campaign,” and we truly value the opportunity to expand the discussion. The points raised contribute to a deeper understanding of our work and its implications for clinical practice.

As explicitly acknowledged in our manuscript, retrospective studies inherently present limitations in terms of data completeness and statistical analyses. We discussed these aspects in detail, including the constraints of the SIRVA registry, the impossibility of accounting for intercurrent infections or vaccinations performed outside our region, and the fact that some apparently incomplete vaccination cycles may have been completed prior to our observation window. Furthermore, we highlighted that the low number of post-vaccination reactions would not allow adequately powered inferential analyses, which led us to favour descriptive statistics in this context.

It is important to emphasise that our study deliberately targeted patients at *high allergic risk*, rather than the general population. Patient referral to our centre therefore does not represent selection bias, but rather an intentional focus on those who required allergological assessment to guarantee safe vaccination. The findings are thus directly relevant to similar high-risk clinical settings.

We particularly appreciate that the correspondence underscores 2 key messages of our study: the very low rate of true allergic reactions to COVID-19 vaccines (0.4%), even in a high-risk population, and the pivotal role of risk assessment and clear physician–patient communication in promoting safe immunisation. The fact that most patients tolerated subsequent doses without major adverse events provides strong evidence supporting this approach.

Finally, regarding skin testing, we agree that such tests have limited predictive value. Their role remains primarily diagnostic in patients with a clinical history suggestive of IgE-mediated reactions. Nonetheless, they may also contribute indirectly to patient reassurance, thereby facilitating adherence to vaccination. Prospective studies are warranted to further clarify this aspect.

In conclusion, we thank the reviewer for their constructive remarks, which enrich the discussion and strengthen the scientific dialogue. We reaffirm that, although the limitations of our study were clearly acknowledged, the main message remains robust: the vast majority of high-risk allergic patients can be safely vaccinated against SARS-CoV-2 following appropriate allergological assessment.

Sincerely,

The Authors

